# Delivery of a functional anti-trypanosome Nanobody in different tsetse fly tissues via a bacterial symbiont, *Sodalis glossinidius*

**DOI:** 10.1186/s12934-014-0156-6

**Published:** 2014-11-07

**Authors:** Linda De Vooght, Guy Caljon, Karin De Ridder, Jan Van Den Abbeele

**Affiliations:** Department of Biomedical Sciences, Unit of Veterinary Protozoology, Institute of Tropical Medicine Antwerp (ITM), Antwerp, Belgium; Unit of Cellular and Molecular Immunology, Vrije Universiteit Brussel, Brussels, Belgium; Laboratory of Myeloid Cell Immunology, VIB, Brussels, Belgium; Department of Physiology, Laboratory of Zoophysiology, University of Ghent, B-9000 Ghent, Belgium

**Keywords:** (3–10), *Sodalis glossinidius*, Symbiont, Paratransgenesis, Recombinant, *Glossina*, Delivery, Functional, Nanobody, *In vivo*, Midgut

## Abstract

**Background:**

*Sodalis glossinidius*, a vertically transmitted microbial symbiont of the tsetse fly, is currently considered as a potential delivery system for anti-trypanosomal components that reduce or eliminate the capability of the tsetse fly host to transmit parasitic trypanosomes, an approach also known as paratransgenesis. An essential step in developing paratransgenic tsetse is the stable colonization of adult flies and their progeny with recombinant *Sodalis* bacteria, expressing trypanocidal effector molecules in tissues where the parasite resides.

**Results:**

In this study, *Sodalis* was tested for its ability to deliver functional anti-trypanosome nanobodies (Nbs) in *Glossina morsitans morsitans*. We characterized the *in vitro* and *in vivo* stability of recombinant *Sodalis* (rec*Sodalis*) expressing a potent trypanolytic nanobody, i.e. Nb_An46. We show that rec*Sodalis* is competitive with WT *Sodalis* in *in vivo* conditions and that tsetse flies transiently cleared of their endogenous WT *Sodalis* population can be successfully repopulated with rec*Sodalis* at high densities. In addition, vertical transmission to the offspring was observed. Finally, we demonstrated that rec*Sodalis* expressed significant levels (ng range) of functional Nb_An46 in different tsetse fly tissues, including the midgut where an important developmental stage of the trypanosome parasite occurs.

**Conclusions:**

We demonstrated the proof-of-concept that the *Sodalis* symbiont can be genetically engineered to express and release significant amounts of functional anti-trypanosome Nbs in different tissues of the tsetse fly. The application of this innovative concept of using pathogen-targeting nanobodies delivered by insect symbiotic bacteria could be extended to other vector-pathogen systems.

**Electronic supplementary material:**

The online version of this article (doi:10.1186/s12934-014-0156-6) contains supplementary material, which is available to authorized users.

## Background

The contemporary response to vector-borne infectious diseases still mainly relies on low-technology interventions, with a major emphasis on vector control through the use of insecticides. As vector-borne diseases continue to present significant threats to human, animal and plant health, there is an urgent need to develop control efforts that remain effective over time. Genetically modified disease vectors that are rendered resistant (refractory) to pathogen transmission can provide unique tools for developing new or complementing existing control strategies (reviewed by [1]). Paratransgenesis is one such approach that aims to reduce vector competence by genetically modifying symbionts of disease vectors and has been demonstrated for *Rhodnius prolixus*, the triatomine vector of Chagas disease [2] and *Anopheles gambiae* [3,4], the principal malaria vector in Africa.

A paratransgenic approach in tsetse flies, the sole vector of major African trypanosome parasites (*T. brucei* sp. and *T. congolense*), is of high interest since tsetse flies are not amenable to germ-line transformation due to their viviparous reproductive biology (intrauterine development and parturition of live offspring [5]). The tsetse fly harbors a natural commensal bacterium i.e. *Sodalis glossinidius*, which is ideally suited as a paratransgenic platform organism since it i) resides in different tsetse tissues that are in close proximity to pathogenic trypanosomes (e.g. midgut) [6]; ii) can be cultured and genetically modified *in vitro* [7,8]; iii) is maternally transmitted to the offspring [9] and iv) is restricted to the tsetse host niche ensuring that this symbiont is a safe candidate for use in the paratransgenic strategy [10].

The characteristics of the selected effector molecule will largely determine the efficacy and specificity of this paratransgenesis approach. Low specificity will generally result in bystander effects that could have a direct or indirect fitness cost for the arthropod. The use of highly specific compounds from the adaptive immune system of vertebrates such as antibody derived fragments is likely to enable highly specific effects without conferring a selective disadvantage to the (para)transgenic arthropods [11]. Nanobodies® (Nbs), representing the smallest known intact antigen-binding fragments derived from camelid heavy-chain only antibodies (HCAbs) [12], are therefore considered as excellent candidates to increase the immune competence of tsetse. Nanobodies targeting distinct epitopes of the variant-specific surface glycoprotein (VSG), abundantly present on the surface of bloodstream trypanosomes have already been identified, some of which were shown to exert direct *in vitro* and *in vivo* trypanolytic activity by interfering with the complex endocytotic machinery that is organized in the flagellar pocket of this parasite [13]. Recently, we developed recombinant *Sodalis* (rec*Sodalis*) strains expressing functional anti-trypanosome nanobodies. These strains were shown to release considerable amounts of functional anti-trypanosome Nbs to the extracellular culture environment and to have no negative effects on the bacterium in an *in vitro* context [14].

Another prerequisite in developing paratransgenic tsetse flies is the development of a methodology that allows the stable repopulation of tsetse flies with rec*Sodalis* strains expressing trypanosome-interfering proteins in insect tissues where trypanosome parasites reside. Previously, rec*Sodalis* expressing green fluorescent protein (GFP) has been successfully introduced into tsetse through thoracic microinjection [6]. Here, GFP-expressing *Sodalis* was found to be present in the haemolymph and gut tissues of injected females and their progeny. However, to date no studies have focused on the densities whereby genetically modified *Sodalis*, expressing heterologous genes, are maintained within the fly and the efficiency of their transmission to the offspring.

In this study, a rec*Sodalis* strain was tested for its ability to deliver functional anti-trypanosome Nbs in the tsetse fly *Glossina morsitans morsitans*. The *in vivo* long-term stability of the recombinant strain and transmission to the progeny was measured using a quantitative PCR (qPCR) analysis. We show that rec*Sodalis* expressing a potent trypanolytic nanobody, i.e. Nb_An46, was stably maintained *in vivo* only when the WT *Sodalis* population was significantly reduced (>95% of the normal population) prior to rec*Sodalis* introduction. Furthermore, we demonstrated that significant levels of functional Nb_An46 accumulated in different fly tissues, including the midgut where an important developmental stage of the trypanosome parasite occurs.

## Results

### *In vitro* culture characteristics of recombinant *Sodalis* expressing Nb_An46

**Figure 1:**
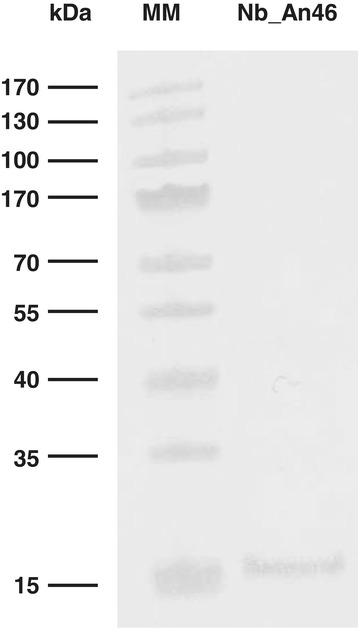
**Qualitative analysis of extracellular Nb_An46 expressed by** ***Sod*** **_pFliCpelBNb46** ***fliC in vitro*** **.** The extracellular expression of Nb_An46 was analyzed by immunoblotting of the medium supernatant using an anti-His antibody (1:1000 Serotec) for detection. Presented data are representative for at least three independent experiments. The PageRuler 10–170 kDa prestained protein ladder (Fermentas) was used as a molecular size marker (MM).

**Figure 2:**
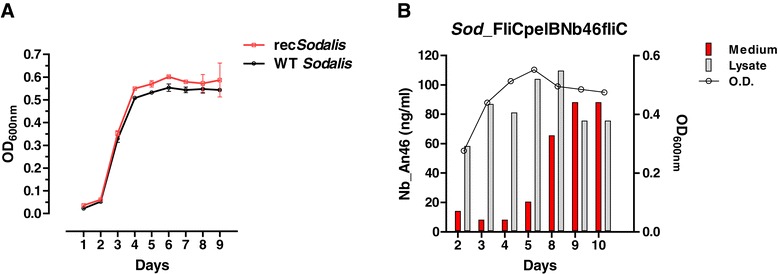
***In vitro*** **characteristics of rec** ***Sodalis*** **expressing Nb_An46. A)** Growth curve analysis of WT *Sodalis* and *Sod*_pFliCpelBNb46*fliC*. The error bars show the ± SD of two biological replicates. Samples were taken every 24 h. **B)** ELISA-based Nb_An46 quantitation (bar-chart) of the intra- and extracellular nanobody concentration produced by *Sod*_pFliCpelBNb46*fliC* at selected time points in relation to bacterial cell density (OD_600nm_, solid line) using a 6 × His tag specific detection antibody. Values are presented as ng recombinant protein per ml culture medium.

Prior to the introduction of rec*Sodalis* into experimental flies, the Nb expression profile, *in vitro* growth rate and plasmid stability of rec*Sodalis* expressing a FliCpelBNb_An46 fusion protein (*Sod*:FliCpelBNb46*fliC*) was established. Extracellular Nb_An46 expression was confirmed by Western blot analysis of supernatant from cultures grown to the beginning of stationary phase (OD_600_ 0.5-0.6) (Figure 1). Nb_An46 expression and release was quantified at different time points during bacterial growth over a 10-day period by measuring the concentration of active Nb in the whole cell lysate and culture medium using a VSG-binding ELISA assay. Functional Nb_An46 was expressed from day 2 onwards and accumulated in the culture medium to a concentration of 88 ng/ml by day 10 (Figure 2B). RecS*odalis* showed normal growth kinetics (Figure 2A) with cell population doubling times comparable to a WT *Sodalis* strain i.e., 15.0 hrs and 14.8 hrs respectively. The number of plasmid copies per cell was estimated to be approximately 20 during the lag and exponential phases of *Sod*:FliCpelBNb46*fliC* grown in the presence of antibiotic selection. The stability of the FliCpelBNb46*fliC* plasmid in rec*Sodalis* was measured by maintaining the recombinant bacteria in log phase growth for 27 generations in liquid MM medium in the absence of antibiotic selection. Colony counts on antibiotic-selective plates showed that 94% of the *Sodalis* population remained antibiotic resistant after 27 generations (corresponding to a 2-month test period) (Table 1).

**Table 1:** **Stability of pFliCpelBNb46** ***FliC*** **in** ***Sodalis*** **under nonselective growth conditions**

**Generations (n)**	**Plasmid-containing CFU (%)**
10	**100**
15	**77**
20	**91**
27	**94**

Plasmid stability was expressed as the ratio between the number of colonies formed on MM blood agar plates with (50 μg/ml) and without kanamycin.

### Prior reduction of WT *Sodalis* is a prerequisite for an efficient host colonization with *recSodalis*

**Figure 3:**
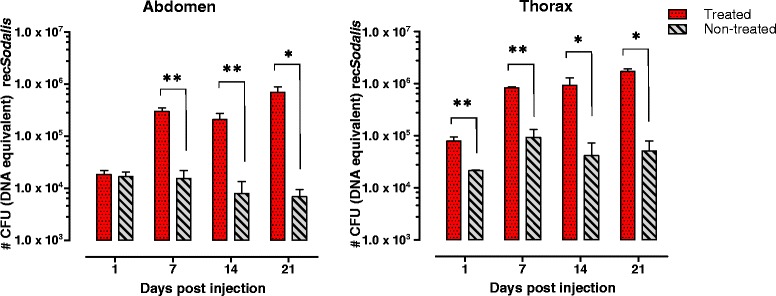
**Number of rec** ***Sodalis*** **CFU (DNA equivalent) in abdomen and thorax of streptozotocin-treated and non-treated male flies injected with 5x10** ^**4**^ **CFU of** ***Sod*** **_pFliCpelBNb46** ***fliC*** **.** Prior to injection, treated flies were given three blood meals supplemented with 20 μg/ml streptozotocin during the first week after emergence, while non-treated flies received normal blood meals. qPCR on the pFliCpelBNb46*FliC* plasmid was used as a means to estimate the number of rec*Sodalis*. The bars represent the mean rec*Sodalis* CFU (DNA equivalent) ± SD present in abdomen and thorax tissues of at least 5 individual flies from each treatment group at the time of sampling. The number of CFU (DNA equivalent) is represented in log scale on the y-axis. P values were calculated using the Mann–Whitney U test (*p <0.05, **p <0.01).

We explored the capability of rec*Sodalis* to colonize the tsetse fly after introduction through intrathoracic injection. We first evaluated the necessity of the prior reduction of the WT *Sodalis* population in tsetse for rec*Sodalis* to establish and colonize its host. In female flies that received 3 streptozotocin supplemented blood meals, the WT *Sodalis* population was reduced by 95% and 88% in abdomen and thorax tissues respectively, compared to flies fed on normal blood. This treatment did not affect the obligatory *Wigglesworthia* symbiont population that mainly resides in the tsetse fly abdomen (Additional file 1: Figure S1). Next, the *in vivo* persistence and growth of the recombinant bacteria in streptozotocin-treated and non-treated flies injected intrathoracically with 5×10^4^ CFU *Sod*_FliCpelBNb46*fliC* was evaluated using qPCR (Figure 3). In streptozotocin-treated flies, rec*Sodalis* was able to proliferate inside its host, reaching densities of 10- and 20-fold the initial injected dose in respectively abdomen and thorax, whereas in non-treated flies the injected rec*Sodalis* population was not able to expand and remained present at its initial density.

### The effect of differential doses of rec*Sodalis* inoculum on tsetse fly colonization and viability

**Figure 4:**
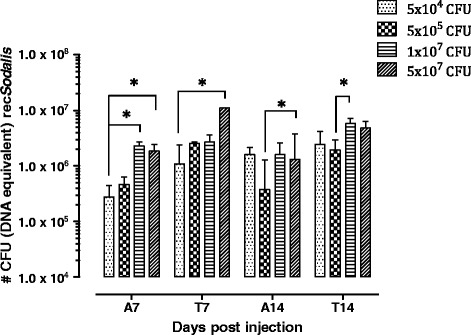
**Number of rec** ***Sodalis*** **CFU (DNA equivalent) present in abdomen (A) and thorax (T) tissues of flies injected with respectively 5×10** ^**4**^ **, 5×10** ^**5**^ **, 1×10** ^**7**^ **and 5×10** ^**7**^ **CFU of** ***Sod*** **_pFliCpelBNb46** ***fliC*** **. qPCR on the pFliCpelBNb46** ***FliC*** **plasmid was used as a means to estimate the number of rec** ***Sodalis*** **.** The bars represent the mean rec*Sodalis* CFU (DNA equivalent) ± SD present in abdomen and thorax tissues of at least 5 individual flies from each treatment group at the time of sampling. The number of CFU (DNA equivalent) is represented in log scale on the y-axis. P values were calculated using the Kruskal–Wallis test followed by Dunn's test for multiple comparison (*p <0.05, **p <0.01).

We determined the optimal dose for rec*Sodalis* injection in terms of host colonization and its viability. For this, streptozotocin-treated male flies were microinjected with either 5 × 10^4^, 5 × 10^5^, 10^7^ and 5 × 10^7^ CFU *Sod*_FliCpelBNb46*fliC* and rec*Sodalis* densities in abdomen and thorax tissues were determined 7 and 14 days post-injection (dpi) using qPCR (Figure 4). Injection of 5 × 10^7^ CFU resulted in an increased fly mortality (up to 59% mortality 14 dpi), whereas limited mortality (≤25% 14 dpi) was observed within the other injection groups. In these groups, rec*Sodalis* was able to repopulate the abdomen and thorax to densities comparable to natural *Sodalis* levels present in WT flies (on average respectively 2.5 × 10^6^ and 1.5 × 10^6^ CFU), demonstrating that a wide range of rec*Sodalis* doses (i.e. 5 × 10^4^ to 1 × 10^7^) are suitable to initiate colonization without affecting fly viability.

### *recSodalis* persists in the tsetse fly and is vertical transmitted to the offspring but at a low extent

**Figure 5:**
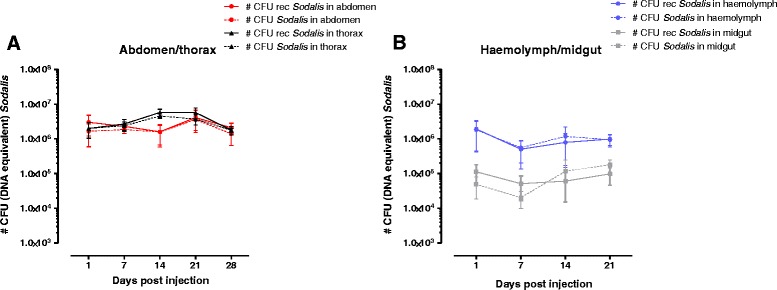
**Evaluating the host colonization properties of rec** ***Sodalis*** **. A)** Number of rec*Sodalis* CFU (DNA equivalent) present in abdomen (solid red line) and thorax (solid black line) of Streptozotocin-treated flies injected with 1x10^7^ CFU of *Sod*_pFliCpelBNb46*fliC* versus the total number of *Sodalis* CFU DNA equivalent (WT + recombinant *Sodalis*) present in abdomen (dashed red line) and thorax tissues (dashed black line). **B)** The number of recombinant and total *Sodalis* CFU (DNA equivalent) present in haemolymph (solid/dashed blue line) and midgut (solid/dashed grey line) were estimated as described above. Data points represent the mean rec*Sodalis* and total *Sodalis* CFU (DNA equivalent) ± SD present in the respective tissues of at least 5 individual flies at the time of sampling. The number of CFU (DNA equivalent) is represented in log scale on the y-axis.

**Figure 6:**
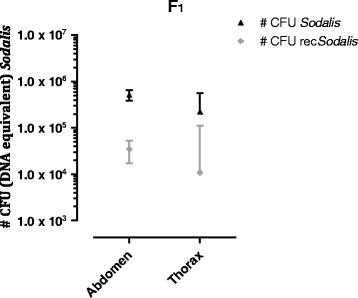
**Transmission of rec** ***Sodalis*** **to the F**_**1**_ **generation was evaluated.** Recombinant and total *Sodalis* numbers were estimated as described above in abdomen and thorax of the F_1_ progeny (freshly emerged) produced by streptozotocin-treated female flies injected with 5 × 10^4^ CFU of *Sod*_pFliCpelBNb46*fliC*. Data points represent the mean rec*Sodalis* and total *Sodalis* CFU (DNA equivalent) ± SD present in the respective tissues of at least 5 individual flies at the time of sampling. The number of CFU (DNA equivalent) is represented in log scale on the y-axis.

The *in vivo* persistence of *Sod*_FliCpelBNb46*fliC* was evaluated by qPCR based estimation of the amount of rec*Sodalis* CFU in abdomen and thorax tissues of streptozotocin-treated male flies injected with 1 × 10^7^ recombinant CFU over a 28 day period (Figure 5A). *Sod*_FliCpelBNb46*fliC* was able to remain present at high densities in abdomen and thorax tissues of experimental flies throughout the course of the 28-day observation period. In flies injected with *Sod*_FliCpelBNb46*fliC*, the entire *Sodalis* population in abdomen and thorax remained recombinant. Next, we evaluated rec*Sodalis* densities in the haemolymph and midgut tissues of flies injected with 1 × 10^7^ recombinant CFU (Figure 5B). *Sod*_FliCpelBNb46*fliC* was able to reach the fly midgut where it persisted at densities between 5 × 10^4^ and 1 × 10^5^ CFU (DNA equivalent) throughout the 21-day observation period. In these flies the obligatory *Wigglesworthia* symbiont population was not affected by the presence of *Sod*_FliCpelBNb46*fliC* (Additional file 1: Figure S2) nor did we observe any effect upon the fecundity of female rec*Sodalis* colonized flies. Transmission dynamics of the recombinant bacteria to the F_1_ progeny was evaluated by qPCR. *Sod*_FliCpelBNb46*fliC* was transmitted to the F_1_ generation, although non-plasmid containing *Sodalis* were dominant in these flies. Indeed, *Sod*_FliCpelBNb46*fliC* constituted only 7 and 5% of the entire *Sodalis* population in respectively abdomen and thorax (Figure 6).

### Functional Nb_An46 is expressed in different tsetse fly tissues

**Figure 7:**
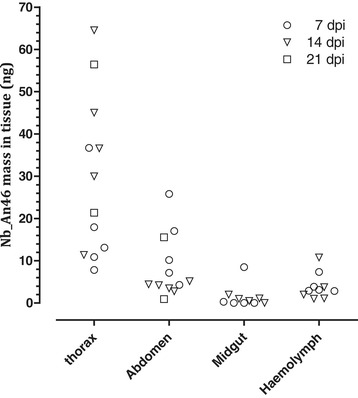
**ELISA based quantitation of the amount of Nb_An46 present in haemolymph, abdomen, thorax and midgut of streptozotocin-treated flies injected with 1×10** ^**7**^ **CFU of** ***Sod*** **_pFliCpelBNb46** ***fliC*** **at selected time points (○:7 dpi, ▽: 14 dpi, □: 7 dpi) using a 6 × His tag specific detection antibody.** Values are presented as ng nanobody per whole tissue extract or 1 μl haemolymph.

Nb_An46 expression in flies injected with 1 × 10^7^*Sod*_FliCpelBNb46*fliC* CFU was quantified using a VSG-binding ELISA. Nanobody concentrations were determined at different time points post-injection in whole abdomen and thorax extracts, haemolymph and midgut (Figure 7). Functional Nb_An46 accumulated in haemolymph and thorax samples of injected flies over time, indicating a continuous transgene expression by rec*Sodalis* in these tissues. In the thorax the Nb_An46 concentration increased from 22 ng on day 14 post-injection to 35 ng on day 21. Although significant quantities of active Nb_An46 were detected in abdomen and midgut using a VSG-binding ELISA, the accuracy of this quantitation could have been hampered by the abundant presence of proteolytic enzymes in the tsetse fly gut, probably resulting in fast degradation of the nanobodies in the tissue homogenates and possibly resulting in the underestimation of the active Nb content of the non-digestive part of the tsetse fly gut.

## Discussion

The aim of this study was to develop a functional *Sodalis*-based shuttle system that is able to deliver and express anti-trypanosome nanobodies within the tsetse fly. In this study a recombinant *Sodalis* strain was used expressing a potent trypanolytic nanobody Nb_An46 through a plasmid-based expression system. The functionality of the system was assessed by monitoring if rec*Sodalis* bacteria can (1) establish a stable population in the tsetse fly over time, (2) express significant levels of active anti-trypanosome nanobodies in the tsetse fly and (3) be efficiently transferred to the offspring.

Although the growth characteristics of Nb-expressing rec*Sodalis* were shown to be similar with those of the WT *Sodalis* in *in vitro* culture conditions it is plausible that in the tsetse fly *in vivo* environment the endogenous WT bacteria could have a competitive advantage over the introduced recombinant bacteria. Our results clearly suggest that rec*Sodalis* is competitive with WT *Sodalis* in *in vivo* conditions, however the maximal total *Sodalis* density in the inner tsetse fly environment seems to be limited to approximately 5×10^6^ CFU. Indeed, only when the existing WT *Sodalis* population is priorly reduced (>95%) by a selective antibiotic treatment, the introduced rec*Sodalis* population is able to maintain and proliferate to comparable density levels observed for wildtype *Sodalis* in normal tsetse flies. This rec*Sodalis* population is then able to outgrow the WT population which remains present at low density. In contrast, in non-treated flies rec*Sodalis* is not able to displace the WT *Sodalis* population but remains present at low density, confirming the importance of creating a WT *Sodalis* deprived niche that allows rec*Sodalis* to proliferate inside its host.

An important factor when using episomally located plasmids in a paratransgenic system is the persistence of these recombinant strains in the tsetse fly in the absence of antibiotic selection as premature loss of expression due to plasmid instability would not be desirable. Therefore, we assessed the stability of the recombinant strains during *in vitro* culture maintenance and after re-introduction in the tsetse fly. Results from the bacterial plate assay showed that in the absence of selection pressure in the culture medium the FliCpelBNb46*fliC* plasmid proved to be stably maintained with 94% of the *Sodalis* population retaining the plasmid after 27 generations. This strong long-term plasmid retention in rec*Sodalis* was not always observed as for another strain (containing the FliCpelBNb33*fliC* plasmid) a complete plasmid loss in the total *Sodalis* population after 20 generations in culture. We postulate that the strong persistence of the FliCpelBNb46*fliC* plasmid in the rec*Sodalis* culture in the absence of a selection pressure is related to the high plasmid copy number that is observed in this strain and which was 20 fold higher than in the unstable *Sod*_FliCpelBNb33*fliC* strain.

*Sod*_FliCpelBNb46*fliC* remained present at high densities in the fly throughout the 28 day observation period. Furthermore, rec*Sodalis* was able to disseminate into the digestive tract reaching densities comparable to those in flies harboring WT symbionts. Furthermore, the majority of the total *Sodalis* population remained recombinant for the duration of the experiment indicating that the high plasmid stability that was observed in the *in vitro* culture is also present in the *in vivo* tsetse fly environment.

Although transmission to the F_1_ generation was observed, this seemed to be highly inefficient as only a small percentage of the total *Sodalis* population was found to be recombinant. This could be due to plasmid loss by rec*Sodalis* during the 30-day pupal stage or the inability of rec*Sodalis* to efficiently colonize the milk glands upon injection in the adult female fly which is a prerequisite for rec*Sodalis* transmission to the intra-uterine larvae through the milk secretion. Indeed analysis of transcripts encoding *Sodalis* motility genes, *fliC* and *motA*, and cell invasion genes, *invA1* and *invA2*, are up regulated in the larval and early pupal stages, and not in adult tsetse flies [15]. These results indicate that the Type-III secretion system and flagellum may be important for the transmission and establishment of symbiont infections in the intra-uterine progeny. Our results clearly indicate that for a successful use of *Sodalis* as paratransgenic vehicle in the tsetse fly several methodologies should still be improved allowing i) the generation of more stable rec*Sodalis* i.e. through an efficient methodology to insert exogenous DNA directly into the bacterial genome and ii) a more efficient transfer of the rec*Sodalis* to the next generations.

Rec*Sodalis* was found to continuously express functional nanobody in the tsetse fly as indicated by the Nb_An46 accumulation in the haemolymph over time. Abdomen and midgut extracts from the majority of insects that carried the recombinant nanobody-producing bacteria were positive in the ELISA, indicating the presence of functional Nb in these tissues. However the abundant presence of proteolytic enzymes in the tsetse fly midgut probably interfered with detection, prohibiting accurate quantification of the Nb expression in this tissue. These results suggest the benefit of lowering the susceptibility of potential effector proteins to proteolytic degradation especially when they have to be functional in a digestive tissue such as the tsetse fly midgut. Nbs have the advantage that they can be mutagenized and selected for increased proteolytic stability [16].

From our experimental data, the expected levels of *in vivo* Nb expression are in the lower ng range. However, these levels of Nb expression should be sufficient to interfere with trypanosome development given that an infective blood meal of a tsetse fly in nature is estimated to contain around 10^3^ parasites as the average parasitaemia in *T. brucei* infected cattle fluctuates from 1.5 × 10^5^ parasites per ml during the acute phase of infection to 5 × 10^4^ parasites per ml during the chronic phase [17] and *in vitro* studies have shown that nanomolar concentrations of trypanolytic Nbs are sufficient to saturate >95% of the surface VSG molecules and to provoke efficient trypanosome lysis [13,18]. Indeed, since a VSG:Nb molar ratio of 1:20 is sufficient to cause efficient lysis, 5 ng of Nb_An46 should be adequate to efficiently lyse 10^3^ parasites.

## Conclusion

These data are the first to show the potential of *Sodalis* as a delivery system for anti-trypanosome effector molecules in tsetse fly tissues relevant for trypanosome development. Given the ability of recombinant *S. glossinidius* to efficiently establish in different tsetse fly tissues at high densities and their capacity to release significant levels of functional anti-trypanosome Nbs in tissues were trypanosomes reside, the foundation has been laid for further exploration of the inhibitory effect on trypanosome development in the tsetse fly. Moreover, a paratransgenic approach using *Sodalis* to deliver Nbs that target the trypanosome-tsetse fly crosstalk could open a new avenue to unravel the molecular determinants of this specific parasite-vector association.

## Material and methods

### Insects, bacterial strains and culture conditions

*G. morsitans morsitans* (Westwood) from the colony at the Institute of Tropical Medicine Antwerp (ITM), originated from pupae collected in Kariba (Zimbabwe) and Handeni (Tanzania), were used in all experiments. Flies, maintained at 26°C and 65% relative humidity, were fed 3 days per week with defibrinated bovine blood using an artificial membrane system. *Sodalis glossinidius* strains used in this study were isolated from the haemolymph of surface-sterilized tsetse flies from the colony maintained at ITM. Cultures were maintained *in vitro* at 27°C in liquid Mitsuhashi-Maramorosch (MM) insect medium (PromoCell) supplemented with 10% (v/v) heat-inactivated fetal bovine serum (FBS). Where appropriate, selection antibiotics were added to the media at the following concentrations: 100 μg/ml of ampicillin or 50 μg/ml of kanamycin. Flies used in this study were maintained at 26°C and 65% relative humidity, and fed 3 days per week with defibrinated bovine blood using an artificial membrane system.

### Plasmid constructs

In this study the pFliCpelBNb46*fliC* plasmid was used in which a 6 × His tagged *Nb*_*An46* gene was fused to two secretion signals (FliC and pelB) and cloned into the multiple cloning site of the pCM66 expression vector. The pFliCpelBNb46*fliC* plasmid was derived from pFliCpelBNb33*fliC* [12] by replacement of *pelBNb*_*An33* between the *Xba*I and *Eco*RI sites by *pelBNb*_*An46* amplified as a *Xba*I-*EcoR*I fragment (465 bp) by PCR from the pHen6C plasmid containing the *pelBNb*_*An46* gene using the following primer set: Nb46_FW, 5’-*TCTAGA*ATGAAATACCTATTGCCTACGG-3’ and Nb46_Rev, 5’-*GAATTC*TTAGTGATGGTGATGGTGGTGGCGGCCGCGTGAGGAGAC-3’ (*Xba*I-*Eco*RI restriction sites are underlined).

### Transformation of *Sodalis glossinidius*

*Sodalis* transformation with the pFliCpelBNb46*fliC* plasmid was conducted using a heat-shock based method as described in [14]. Following transformation, the cells were allowed to recover overnight at 27°C in liquid MM medium prior to plating.

### Growth curve measurements

Logarithmically growing cultures were used to inoculate 25 ml of MM-medium to an optical density at 600 nm (OD_600_) of 0.05. *Sodalis* cultures harboring the FliCpelBNb46*fliC* plasmid were allowed to grow without shaking for the first 24 h, after which they were transferred to a shaking incubator. Samples were taken every 24 h for optical density measurements and Nb protein quantification in culture supernatants and whole cell extracts. The cell population doubling time was calculated from the growth rate during exponential growing phase using the following equation: doubling time (in hours) = h*ln(2)/ln(c2/c1) where c1 is the initial concentration and c2 is the concentration when cultures reached maximum densities.

### Plasmid copy number assay

The plasmid copy number per cell was calculated by dividing the absolute copy number by the number of *Sodalis* CFU present in the culture. The absolute number of plasmid and *Sodalis* CFU present in the *Sod*_FlicpelBNb46*fliC* culture grown in the presence of antibiotic selection (kanamycin 50 μg/ml) was estimated by quantitative real-time PCR (qPCR) on DNA extracted from culture samples taken during the lag and exponential growth phases using the QIAGEN DNeasy extraction kit (QIAGEN). PCR reactions were carried out on a LightCycler™ (Roche Diagnostics, Mannheim, Germany). To estimate the number of *Sodalis* CFU present in the culture, a standard curve was generated using DNA extracts from a serial dilution series (1:10) ranging from 10^7^*Sod*_FlicpelBNb46*fliC* CFU/ml to 10^2^*Sod*_FlicpelBNb46*fliC* CFU/ml. For pFliCpelbNb46*fliC* plasmid number determination a dilution series (1:10) ranging from 10-10^10^ plasmid copies μl^−1^ was prepared to establish a linear standard curve for real-time PCR assays. For *Sodalis*, primers that target a 120-bp region of the single-copy *S. glossinidius exochitinase* gene were used: Qchi_Fw, 5’- TGGGGACAGTACGATGGCAGAGC −3 ; Qchi_Rev, 5’- TCATAGGCGGTCGGGGATAATTGCG -3’. For the plasmid number determination, a 433-bp common region present on both pFliCpelBNb33*fliC* and pFliCpelBNb46*fliC* plasmids was amplified using the following primer set: pCM66_Fw, 5’- CTTGGCCCTCACTGACAG-3’; pCM66_rev, 5’- GCAGCCCTGGTTAAAAAC-3’. qPCR was performed in a 20-μl reaction mixture volume containing 10 μl of 2 × iQ™ SYBR green supermix, 0.3 μM of each primer, template (DNA ) and RNase-free sterile water to a final volume of 20 μl.

### Measuring *in vitro* plasmid stability

The *in vitro* stability of the pFliCpelBNb46*fliC* plasmid in *Sodalis* was measured by maintaining recombinant bacteria in log phase growth for 27 generations in MM without kanamycin selection. Samples were taken every two to four generations and plated on MM blood agar with and without kanamycin. The ratio of the number of colony forming units (CFU) grown on the selective blood agar plate to those grown on the non-selective blood agar was used to determine the percentage of plasmid-carrying cells. This ratio allows to monitor the plasmid stability during subsequent generations in the cultured rec*Sodalis*.

### Western blot analysis

Cells were pelleted from bacterial cultures by centrifugation (15 min, 10000 × g) and the supernatant was clarified from residual bacterial cells by a second centrifugation step. Proteins in the growth medium were precipitated with 10% trichloroacetic acid (TCA) for 1 h on ice. For SDS-PAGE, samples were heat denatured at 95°C in the presence of SDS-PAGE loading buffer containing β-mercaptoethanol and analyzed on a 12% (w/v) polyacrylamide gel (Biorad). Proteins were transferred onto a Hybond C nitrocellulose membrane (Whattman). After overnight blocking with 1% (w/v) bovine serum albumin, the membrane was incubated sequentially with a mouse anti-6 × His-tag IgG1 antibody (1:1000) (Serotec) and a rabbit anti-mouse-IgG antibody (1:1000) (Serotec) conjugated to horseradish peroxidase. In between these successive 2 h incubations, the membrane was washed with PBS-0.1% Tween 20. Thirty minutes after adding the substrate (TMB 1-Component Membrane Peroxidase Substrate, KPL) the reaction was stopped by washing the membrane with water.

### *RecSodalis* introduction into tsetse flies

To evaluate the effect of reducing the WT *Sodalis* population in tsetse on rec*Sodalis* growth inside its host, newly emerged male *G. morsitans morsitans* flies were divided into two experimental groups. The treated group was given three blood meals supplemented with 20 μg/ml streptozotocin while the non-treated group received normal blood meals. Next, treated and non-treated flies were briefly anaesthetized by cold shock and microinjected intrathoracically with a suspension of 5 × 10^4^*Sod*_pFliCpelBNb46*fliC* CFU, using a 5 μl Hamilton 75RN microsyringe with gauge 33 removable electrotapered needles. After injection, males were kept in separate cages and received an antibiotics-free blood meal every 48 h throughout the course of the experiment.

The optimal rec*Sodalis* injection dose was evaluated by injecting streptozotocin treated adult male flies with respectively 5 × 10^4^, 5 × 10^5^, 10^7^ and 5 × 10^7^ CFU *Sod*_pFliCpelBNb46fliC followed by determination of the number of rec*Sodalis* CFU in abdomen and thorax 7 and 14 days post-injection.

To evaluate the *in vivo* persistence of *Sod*_pFliCpelBNb46*fliC*, streptozotocin treated adult male flies were injected intrathoracically with 10^7^*Sod*_pFliCpelBNb46*fliC* CFU. At different time points post-injection i.e. day 1 (teneral), 7, 14, 21 and 28, the number of WT and rec*Sodalis* CFU present in abdomen, thorax, haemolymph and midgut was measured using qPCR. To evaluate the transmission of rec*Sodalis* to the F_1_ progeny, streptozotocin-treated female flies were injected intrathoracically with 5 × 10^4^*Sod*:pFliCpelBNb46*fliC* CFU. Females were allowed to mate with WT males and their offspring (teneral stage) was evaluated for the presence of rec*Sodalis*.

### Measuring *in vivo Sodalis* and *Wigglesworthia* density

At various time points post-injection flies were sacrificed for genomic DNA extraction using the QIAGEN DNeasy extraction kit (QIAGEN). We used a quantitative PCR (qPCR) method for the estimation of the number of WT *Sodalis* and rec*Sodalis* cells in tsetse fly tissues (abdomen, thorax, midgut and haemolymph). For this, triplicate cultures of WT and rec*Sodalis* were serially diluted (10-fold) in PBS to yield a *Sodalis* density ranging from 10^7^ CFU/ml to 10^2^ CFU/ml. DNA was extracted from each *Sodalis* dilution followed by qPCR using primers that amplify a region of the pCM66 plasmid backbone present in rec*Sodalis*: (pCM66_Fw, 5’-CTTGGCCCTCACTGACAG-3’ and pCM66_Rev, 5’- GCAGCCCTGGTTAAAAAC-3’) and primers that target a 120-bp region of the single-copy *Sodalis glossinidius exochitinase* (Qchi) gene to determine the corresponding Ct values. Standard curves were generated by plotting these C_t_ values against the corresponding log of *Sodalis* CFU/ml. This qPCR approach in combination with the *Sodalis* CFU standard curves allowed us to estimate the CFU (DNA equivalent) values present in the tsetse fly tissues of the different experimental fly series. An internal control was included to evaluate the DNA extraction efficiency in all the tissue samples. For this, samples were spiked with 0.4 ng of plasmid DNA (pBAD24_GFP) prior to extraction. qPCR with *gfp*-specific primers (pBAD24_GFP: GFP_Fw, 5’-TGGCCAACACTTGTCACTAC-3’ and GFP_Rev, 5’-AGAAGGACCATGTGGTC-3’) revealed a C_t_ value of approximately 24.5 in all the DNA extracts corresponding to the C_t_ value of the plasmid dilution used to spike to internal control alone demonstrating a uniform highly efficient extraction of the DNA in all the samples.

*Wigglesworthia* quantification was done by amplifying the *thiamine* locus with the following primer set: QthiC_F, 5’-AAGTTATGATAGAAGGACCAGGAC-3’ and QthiC_R, 5’-CCCGGAGCAATATCAGTAGTTAG-3’. All Ct values were normalized to the *G. morsitans morsitans* reference gene β-actin for each sample using the following primer set: β-actin_F, 5’-GGCTTCTGGTCGTACTACT-3’ and β-actin_R, 5’-CCGGACATCACAATGTTGG-3’. For each sample the obtained Ct value of the thiamine gene was normalized to the Ct value of the reference gene (β-actin) by subtracting the β-actin Ct value from the thiamine Ct value to yield a ΔCt value for each sample. ΔCt values were log-transformed and compared using the Mann–Whitney U test to establish differences among treated and control groups. A value of p <0.05 was considered significant in all analyses.

qPCR was performed in a 20-μl reaction mixture volume containing 10 μl of 2 × iQ™ SYBR green supermix, 0.3 μM of each primer, template (genomic DNA, standard) and RNase-free sterile water to a final volume of 20 μl. The amount of fluorescence generated was measured during each amplification cycle using the following program: (i) initial denaturing at 95°C for 3 min; (ii) 40 cycles, with 1 cycle consisting of denaturation at 95°C for 10 s, annealing at 60°C for 10 s, and extension at 72°C for 30 s. All assays were carried out on a LightCycler™ (Roche Diagnostics, Mannheim, Germany) in 96-well format plates in duplicate and were averaged for each sample. For each PCR run, a negative (no-template) control was used to test for false-positive results or contamination.

### Analysis of *in vitro* and *in vivo* Nanobody concentrations using ELISA

i. ***In vitro***. The amount of active Nb_An46 present in cytoplasmic extracts and growth medium was quantified using an optimized nanobody-detection ELISA [19]. For this purpose, Maxisorb 96-well plates (Nunc) were coated overnight (4°C) with 200 ng purified soluble AnTat 1.1 VSG per well in 0.1 M NaHCO_3_, pH 8.2. Residual protein binding sites were blocked for two hours at room temperature with 0.5% bovine serum albumin (BSA) in PBS. Standards and samples were added for 1 h at room temperature. Detection of antigen-bound nanobodies was performed with a mouse anti-6 × His IgG antibody (Serotec) directly conjugated to horseradish peroxidase. Thirty minutes after adding peroxidase substrate, the reaction was stopped with 0.33 M H_2_SO_4_ and the optical density was measured at 450 nm (690 nm was used as reference filter). Protein concentrations were calculated from a standard curve fitted to a four parameter logistic equation using the Ascent software (Labsystems). Samples from the *in vitro Sodalis* culture were taken at the same time points indicated for the growth curve measurements. For each sample, 1 ml of culture media was centrifuged two times (8000 × g) to obtain the extracellular and whole cell fractions. The whole cell extracts were prepared by resuspending the cell pellets in 0.2 ml PBS supplemented with complete protease inhibitor (Roche) followed by sonication at an amplitude of 10 microns for 5 seconds (3 cycles on ice). For quantification of Nb_An46 present in cytoplasmic extracts and growth medium, a standard serial dilution series (1:2) starting from 2500 to 5 ng/ml of purified Nb_An46 was prepared in PBS and MM-medium respectively. MM medium and PBS alone were included as blanks.
ii. ***In vivo***. Quantification of active Nb_An46 present in different tsetse fly tissues samples (abdomen, thorax, midgut and haemolymph) was performed at different time points post-injection. The preparation of the whole tissue extracts by sonication and the Nb-quantification by ELISA were performed as described above. To mimic the sample complexity of the abdomen, thorax, midgut and haemolymph extracts, nanobody standards were prepared in the corresponding tissue extracts from non-injected wildtype flies. The respective tissues from flies harboring WT *Sodalis* were included as blanks.

## Additional file

Additional file 1: Figure S1. The presence of *Wigglesworthia* in abdomen tissues from streptozotocin treated and non-treated male flies. **Figure S2.** The presence of *Wigglesworthi*a in abdomen of male flies injected with 107 CFU of *Sod_*pFliCpelBNb46*fliC* at day 7, 14 and 21 post injection and control flies.

## References

[1] Coutinho-Abreu IV, Zhu KY, Ramalho-Ortigao M (2010). Transgenesis and paratransgenesis to control insect-borne diseases: current status and future challenges. Parasitol Int.

[2] Durvasula RV, Gumbs A, Panackal A, Kruglov O, Aksoy S, Merrifield RB, Richards FF, Beard CB (1997). Prevention of insect-borne disease: an approach using transgenic symbiotic bacteria. Proc Natl Acad Sci U S A.

[3] Fang W, Vega-Rodriguez J, Ghosh AK, Jacobs-Lorena M, Kang A, St Leger RJ (2011). Development of transgenic fungi that kill human malaria parasites in mosquitoes. Science.

[4] Wang S, Ghosh AK, Bongio N, Stebbings KA, Lampe DJ, Jacobs-Lorena M (2012). Fighting malaria with engineered symbiotic bacteria from vector mosquitoes. Proc Natl Acad Sci U S A.

[5] Attardo GM, Guz N, Strickler-Dinglasan P, Aksoy S (2006). Molecular aspects of viviparous reproductive biology of the tsetse fly (*Glossina morsitans morsitans*): Regulation of yolk and milk gland protein synthesis. J Insect Physiol.

[6] Cheng Q, Aksoy S (1999). Tissue tropism, transmission and expression of foreign genes *in vivo* in midgut symbionts of tsetse flies. Insect Mol Biol.

[7] Welburn SC, Maudlin I, Ellis DS (1987). *In vitro* cultivation of rickettsia-like-organisms from *Glossina spp*. Ann Trop Med Parasitol.

[8] Beard CB, O'Neill SL, Mason P, Mandelco L, Woese CR, Tesh RB, Richards FF, Aksoy S (1993). Genetic transformation and phylogeny of bacterial symbionts from tsetse. Insect Mol Biol.

[9] Attardo GM, Lohs C, Heddi A, Alam UH, Yildirim S, Aksoy S (2008). Analysis of milk gland structure and function in *Glossina morsitans*: milk protein production, symbiont populations and fecundity. J Insect Physiol.

[10] Van Den Abbeele J, Bourtzis K, Weiss B, Cordón-Rosales C, Miller W, Abd-Alla AM, Parker A (2013). Enhancing tsetse fly refractoriness to trypanosome infection–a new IAEA coordinated research project. J Invertebr Pathol.

[11] Caljon G, De Vooght L, Van Den Abbeele J (2013). Options for the delivery of Nanobodies as anti-pathogen molecules in arthropod vectors. J Invertebr Pathol.

[12] Hamers-Casterman C, Atarhouch T, Muyldermans S, Robinson G, Hamers C, Songa EB, Bendahman N, Hamers R (1993). Naturally occurring antibodies devoid of light chains. Nature.

[13] Stijlemans B, Caljon G, Natesan SK, Saerens D, Conrath K, Perez-Morga D, Skepper JN, Nikolaou A, Brys L, Pays E, Magez S, Field MC, De Baetselier P, Muyldermans S (2011). High affinity nanobodies against the *Trypanosome brucei* VSG are potent trypanolytic agents that block endocytosis. PLoS Pathog.

[14] De Vooght L, Caljon G, Stijlemans B, De Beatselier P, Coosemans M, Van Den Abbeele J (2012). Expression and extracellular release of a functional anti-trypanosome Nanobody® in *Sodalis glossinidius*, a bacterial symbiont of the tsetse fly. Microb Cell Fact.

[15] Toh H, Weiss BL, Perkin SA, Yamashita A, Oshima K, Hattori M, Aksoy S (2006). Massive genome erosion and functional adaptations provide insights into the symbiotic lifestyle of *Sodalis glossinidius* in the tsetse host. Genome Res.

[16] Harmsen MM, van Solt CB, van Zijderveld-van Bemmel AM, Niewold TA, van Zijderveld FG (2006). Selection and optimization of proteolytically stable llama single-domain antibody fragments for oral immunotherapy. Appl Microbiol Biotechnol.

[17] Van den Bossche P, Ky-Zerbo A, Brandt J, Marcotty T, Geerts S, De DR (2005). Transmissibility of *Trypanosoma brucei* during its development in cattle. Trop Med Int Health.

[18] Caljon G, Stijlemans B, Saerens D, Van Den Abbeele J, Muyldermans S, Magez S, De Baetselier P (2012). Affinity is an important determinant of the anti-trypanosome activity of nanobodies. PLoS Negl Trop Dis.

[19] Caljon G, Caveliers V, Lahoutte T, Stijlemans B, Ghassabeh GH, Van den Abbeele J, Smolders I, De Baetselier P, Michotte Y, Muyldermans S, Magez S, Clinckers R (2012). Using microdialysis to analyse the passage of monovalent nanobodies through the blood–brain barrier. Br J Pharmacol.

