# Supplementary material for: Delivery of a functional anti-trypanosome Nanobody in different tsetse fly tissues via a bacterial symbiont, Sodalis glossinidius
Source: Microb Cell Fact. 2014 Nov 7;13:156. doi: 10.1186/s12934-014-0156-6 (PMC4230353; doi:10.1186/s12934-014-0156-6)

Additional file 1

Figure S1 - The presence of *Wigglesworthia* in abdomen tissues from streptozotocin treated and non-treated male flies.

Treated flies were subjected to 3 blood meals supplemented with streptozotocin 20 µg/ml during the first week posteclosion. Samples were taken at day 7 and 14 after administration of the last streptozotocin supplemented bloodmeal. The bars represent the mean Ct values ± SD of the *Wigglesworthia thiamine* (Qthi) gene and the *G. morsitans morsitans β-actin* (Actin) reference gene in abdomen tissues of at least 8 individual flies from each treatment group at the time of sampling. The mean delta Ct values for the treated and non-treated groups at day 7 and 14 are plotted against the right Y-axis. In abdomen tissues, where the *Wigglesworthia* symbiont mainly resides, no significant difference (p=0.13 on day 7 and p=0.27 on day 14 post streptozotocin treatment) in ΔCt-values was observed between treated and non-treated groups, indicating that *Wigglesworthia* was not affected by the streptozotocin treatment.

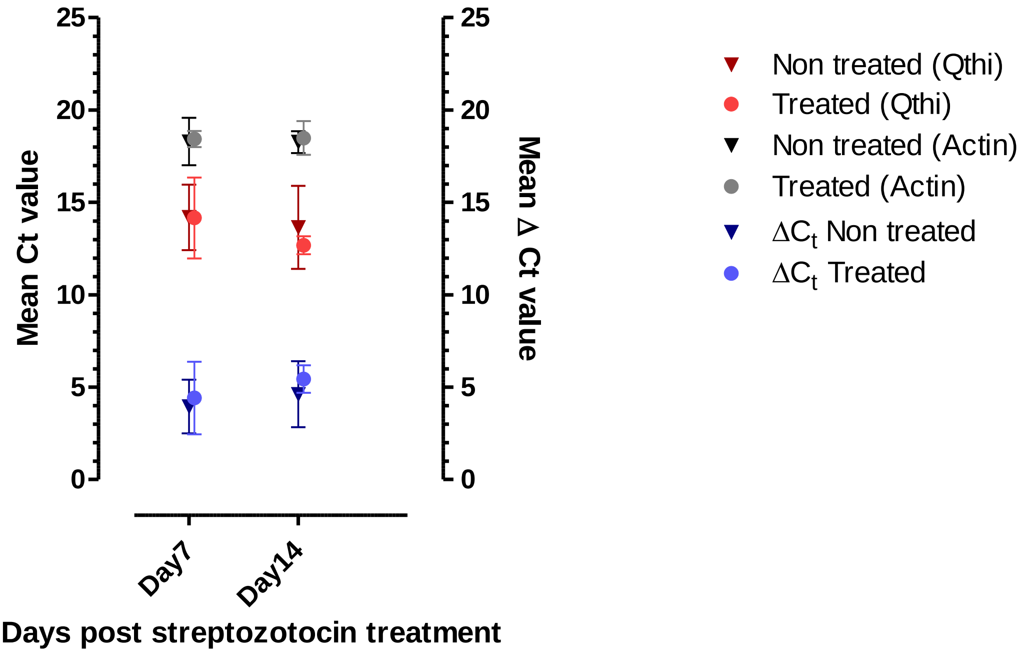

Figure S2 - The presence of *Wigglesworthi*a in abdomen of male flies injected with 10^7 CFU of *Sod*_pFliCpelBNb46*fliC* at day 7, 14 and 21 post injection and control flies.

Data are shown as mean delta Ct values compared to β-actin as a reference gene. In abdomen tissues, where the *Wigglesworthia* symbiont mainly resides, no significant difference ($p=0.63$ on day 7, $p=0.21$ on day 14 and $p=0.8$ on day 21 post-rec*Sodalis* injection) in ΔCt-values was observed between injected and control groups, indicating that *Wigglesworthia* was not affected in streptozotocin-treated flies micro-injected with rec*Sodalis*.

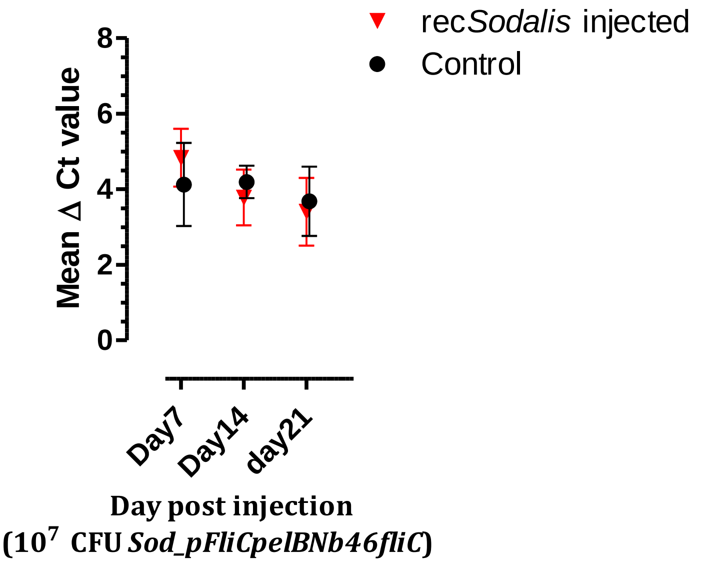

Supplement: Additional file 1: Figure S1. — The presence of Wigglesworthia in abdomen tissues from streptozotocin treated and non-treated male flies. Figure S2. The presence of Wigglesworthia in abdomen of male flies injected with 107 CFU of Sod_pFliCpelBNb46fliC at day 7, 14 and 21 post injection and control flies. [file 12934_2014_156_MOESM1_ESM.pdf]
